# Effectiveness of healthcare worker screening in hospital outbreaks with gram-negative pathogens: a systematic review

**DOI:** 10.1186/s13756-018-0330-4

**Published:** 2018-03-09

**Authors:** Nikos Ulrich, Petra Gastmeier, Ralf-Peter Vonberg

**Affiliations:** 1 Charité - Institute for Hygiene and Environmental Medicine, Charité-University Medicine Berlin, Berlin, Germany; 2 Institute for Medical Microbiology and Hospital Epidemiology, Hannover Medical School, Hannover, Germany

**Keywords:** Outbreak, Gram negative, Health care worker, Screening

## Abstract

**Background:**

Identifying the source of an outbreak is the most crucial aspect of any outbreak investigation. In this review, we address the frequently discussed question of whether (rectal) screening of health care workers (HCWs) should be carried out when dealing with outbreaks caused by gram negative bacteria (GNB).

A systematic search of the medical literature was performed, including the Worldwide Outbreak Database and PubMed. Outbreaks got included if a HCW was the source of the outbreak and the causative pathogen was an *Escherichia coli*, *Klebsiella spp*., *Enterobacter spp*., *Serratia spp*., *Pseudomonas aeruginosa*, or *Acinetobacter baumannii.*

This was true for 25 articles in which there were 1196 (2.1%) outbreaks due to GNB, thereof 14 HCWs who were permanently colonized by the outbreak strain. Rectal screening of HCWs was helpful in only 2 of the 1196 (0.2%) outbreaks. Instead, the hands of HCWs served as a reservoir for the outbreak strain in at least 7 articles – especially when they suffered from onychomycosis or used artificial fingernails or rings.

**Conclusion:**

Due to very weak evidence, we do not recommend rectal screening of HCWs in an outbreak situation with GNB. However, besides a critical review of hand hygiene habits, it might be useful to examine the hands of staff carefully. This measure is cheap, quick to perform, and seems to be quite effective.

**Electronic supplementary material:**

The online version of this article (10.1186/s13756-018-0330-4) contains supplementary material, which is available to authorized users.

## Background

The value of screening healthcare workers (HCWs) during an investigation of a nosocomial outbreak is yet unclear. There is some evidence, that this may be a beneficial measure when dealing with *Staphylococcus aureus* as the causative agent, if there is a clear epidemiological link between an individual HCW and the occurrence of *Staphylococcus aureus* infections among patients while no other outbreak source seems likely [1]. This is because decolonization strategies exist for this particular pathogen. However, even in Methicillin-resistant *Staphylococcus aureus* (MRSA) outbreaks, HCWs are rarely identified as its cause: A systematic review on 191 nosocomial MRSA outbreaks showed that in only 11, HCWs were the outbreak’s source. Among them were 3 HCWs (1.6% of all nosocomial MRSA outbreaks) that were asymptomatic carriers who would normally have been missed had the screening of staff not taken place. The remaining 8 HCWs had MRSA infections themselves, which means that screening does not give any additional information [2]. Personnel screening can be useful in certain situations and certain pathogens. In a nosocomial Outbreaks in the USA in 1996 and 1997 HCW screening was initiated after postpartum Group A streptococcus (GAS) was observed in 9 patients. After a case-control study, they found that exposure to one HCW was strongly associated with infection of GAS. As a consequence, screening of 198 HCWs resulted in three positive tests. However, only one HCW’s test culture matched the outbreak strain. The HCW had a rectal colonisation. After antibiotic treatment of the HCW there were no additional cases in the hospital [3].

Even less is known about this matter when dealing with nosocomial outbreaks caused by gram negative bacteria (GNB). National guidelines differ in terms of whether HCW screening should be applied or not. Whereas the ESCMID (European Society of Clinical Microbiology and Infectious Diseases) for example recommends HCW screening for outbreaks with some pathogens, the PHE (Public Health England) CPE guidelines do not recommend HCW screening [4]. A recently published systematic review of outbreaks due to extended spectrum beta-lactamase (ESBL)-producing *Enterobacteriaceae* in neonatal intensive care units (NICU) mentioned screening of staff and environmental screening as the main infection control procedures: A total of 75 articles were included, 60 of them provided information regarding infection control measures, thereof 29 outbreak reports, in which screening of HCW was performed [5].

Screening of HCWs during an outbreak investigation may be difficult because the detection of the outbreak strain in swabs from HCWs does not necessarily prove the direction of pathogen spread (chicken vs. egg). Therefore, there is a need for a compre-hensive analysis of this measure, in particular nosocomial outbreaks caused by GNB.

## Methods

### Databases

**Fig. 1:**
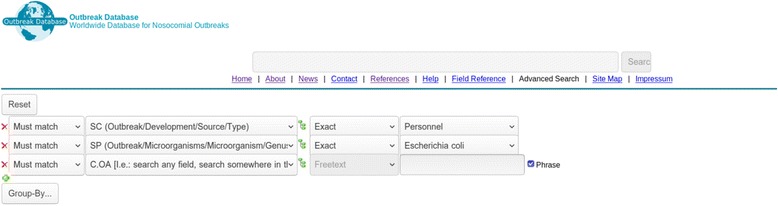
Screenshot of the electronic search strategy in the Worldwide Outbreak Database

The Worldwide Outbreak Database (www.outbreak-database.com) is the largest collection of nosocomial outbreaks. Currently it contains more than 3500 systematically filed outbreak reports as published in the medical literature between 1972 and today [6, 7]. The advanced search mode allows the user of this database to combine parameters of interest. For this systematic review, the first parameter “SC (Outbreak/Development/Source/Type)” was set to “Personnel” and consecutively the second parameter “SP (Outbreak/Microorganisms/Microorganism/Genus/Species/Name)” was set to either one of the following six types of nosocomial pathogens: *Escherichia coli, Klebsiella spp., Enterobacter spp., Serratia spp., Pseudomonas aeruginosa, and Acinetobacter baumannii* (Fig. 1) resulting in 6 separate searches (one for each pathogen). We thereby only retrieved articles in which Personnel was the source of the outbreak of the specified pathogen. The database was accessed on July 25th of 2017.

In addition, PubMed (www.ncbi.nlm.nih.gov/pubmed) was searched on the same day using the following search algorithm: ((nosocomial) AND (outbreak OR epidemic)) AND (Escherichia OR Klebsiella OR Enterobacter OR Serratia OR Pseudomonas OR Acinetobacter).

Finally, all reference lists of the retrieved articles were checked for any further relevant articles that might have been missed.

### Inclusion and exclusion criteria

Outbreak reports were included, if a member of hospital staff was considered the probable or proven cause of an outbreak caused by one of the above-mentioned pathogens. There were no restrictions with respect to the type of medical department, geographical origin or date. Articles in German and English were considered for this review.

The majority of these species are part of the human physiological gut flora. That is why screening for GNB is normally carried out by rectal swabs, culturing of stool samples, or perianal screening [8], although colonisation may also occur at other body sites [9]. However, hands of HCWs are considered the most important vector for the transmission of nosocomial pathogens and, thus, often become part of screening programs, too (although the flora on hands very much depends upon the compliance to hand hygiene). Taking those facts into consideration, it is justifiable to differentiate between gut colonisation, temporary hand contamination, and permanent hand colonisation and/or infection. This systematic review focuses on the epidemiologically most relevant group, HCWs who carry GNB on their hands permanently.

### Data extraction

The following items were extracted from each outbreak report: country, department, risk factors, type of infections, duration, pathogen species, the number of patients involved, mortality, location of colonisation and/or infection of personnel (if applicable), typing method, and typing results.

N.U. carried out the literature review. P.G. independently accessed the content of included articles. Any deviations were discussed and settled by discussion of all three authors (N.U., P.G. and R.P.V.).

## Results

The Worldwide Outbreak Database contained 3551 outbreaks overall, of which 1196 outbreaks were caused by the aforementioned GNB (*E. coli*: 80; *Klebsiella spp.*: 318; *Enterobacter spp.*: 121; *Serratia spp.*: 175; *P. aeruginosa*: 255; *A. baumannii*: 247). The PubMed search, as described, retrieved 1002 potentially relevant articles however all of those were already covered by the primary search of the Worldwide Outbreak Database. No further references were found by checking the citation lists.

**Fig. 2:**
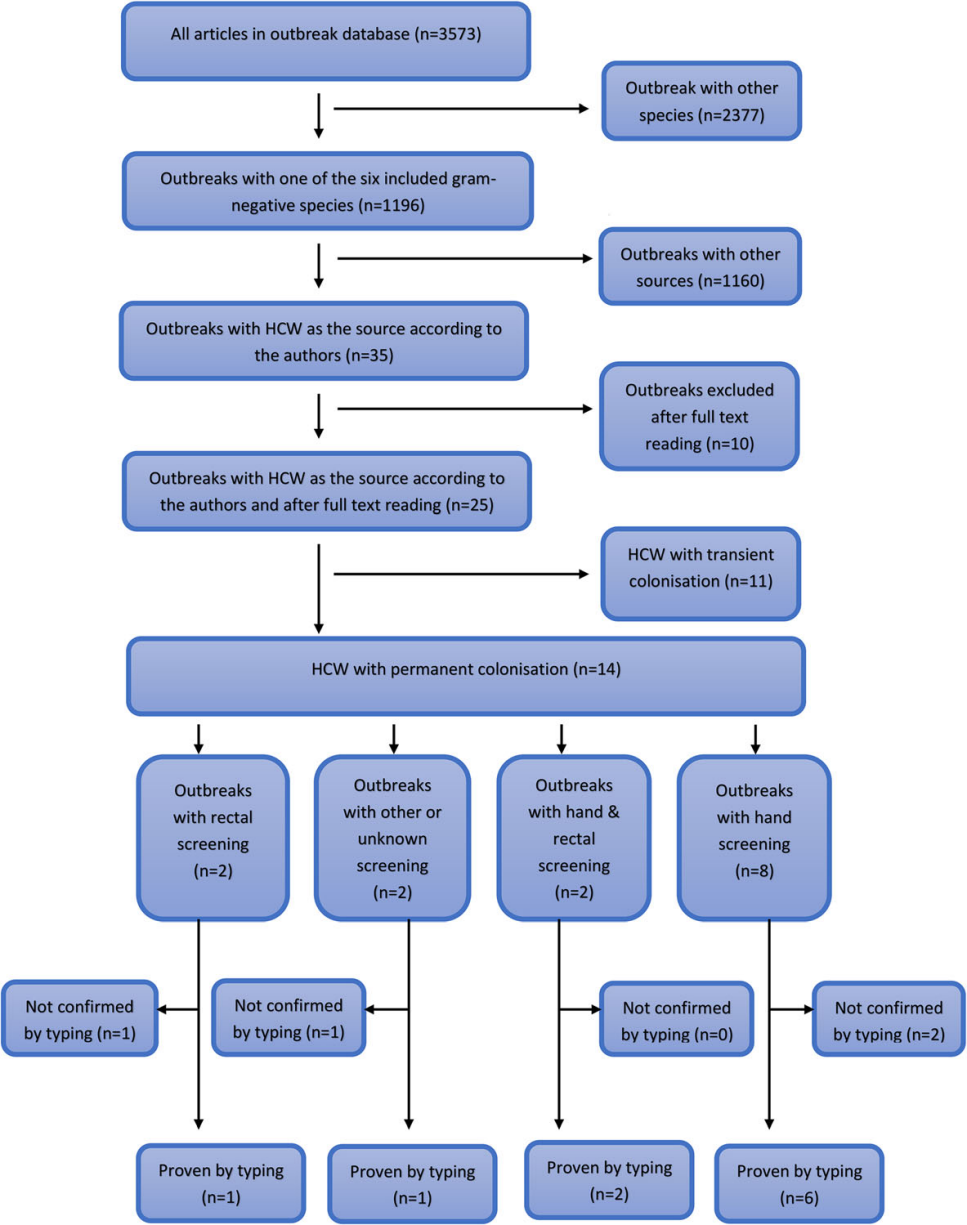
Prisma flow diagram according to the search protocol

All articles in the Worldwide Outbreak Database are already filed in a standardized manner (which allowed the stratified search in the first place) including stratification by the outbreak’s source. HCWs were considered to be the actual source of the nosocomial outbreak in 35 [10–44] of the 1002 reports (= 3.6%) by the authors of the articles. However, we had to exclude 10 [10, 13, 16, 18, 19, 28, 30, 35, 42, 44] articles after full text reading because they either provided insufficient evidence, had a general lack of data, took place at facilities other than hospitals (e.g., long term care facilities), or did not focus on GNB in particular. The colonisation status of the HCWs in the remaining 25 articles were either transient (11 articles) or permanent (14 articles). In 10 of the 14 permanently colonised HCWs, screening of hands was performed and were finally included in this systematic review [20, 25, 27, 29, 31, 32, 34, 36, 39, 41]. Figure 2 shows the corresponding flow chart of the inclusion algorithm of the literature.

The 10 included articles were published between 1970 and 2005 and involved at least 164 patients. Neonatology/nursery and surgery were affected by those events (Table 1). The most frequently observed infections in those 10 outbreaks were bloodstream infections (*n* = 19), wound infections (*n* = 15) and infections of the mediastinum and the pleural cavity (*n* = 10) (for further information see Additional file 1). “Non-physiological conditions of the fingers and hands”, such as onychomycosis, use of artificial nails, and rings, played important roles in 7 of the 10 outbreaks (Table 1).

**Table 1:** Nosocomial outbreaks caused by health care workers involving gram negative bacteria

Autor, year, reference	Pathogen	Department	# Patients	Microbiologically proven	Pathogen identified by rectal swabs	Location at personnel (colonisation and/or infection)
Falcao et al. 1972 [32]	*Pseudomonas aeruginosa*	Nursery	9	yes	yes	• faeces
Passaro et al.1997 [41]	*Serratia marcescens*	Surgery	7	yes	no	• hands (artificial nails)
Moolenaar et al. 2000 [36]	*Pseudomonas aeruginosa*	NICU	46	yes	no	• hands (fingernails)
McNeil et al. 2001 [34]	*Pseudomonas aeruginosa*	Surgery	16	yes	no	• hands (onychomycosis and fingernails)
Taneja et al., 2003 [20]	*Escherichia coli* (ETEC)	NICU	16	yes	yes	• hands • faeces
Zawacki et al. 2004 [31]	*Pseudomonas aeruginosa*	NICU	5	yes	no	• hands • ear
Gupta et al. 2004 [25]	*Klebsiella pneumoniae* (ESBL)	NICU	19	yes	no	• hands (artificial nails)
Boszczowski et al. 2005 [27]	*Klebsiella pneumoniae* (ESBL)	NICU	4	yes	no	• hands (onychomykosis)
Jepson et al. 2006 [39]	*Serratia marcescens*	Surgery	6	yes	no	• hands (ring)
Cassettari et al. 2009 [29]	*Klebsiella pneumoniae* (ESBL)	Neonatal Intermediate Care Unit	36	yes	no	• hands (onychomycotic lesions)

*ETEC* enterotoxigenic *Escherichia coli*, *ESBL* extended spectrum beta-lactamase, *NICU* neonatal intensive care unit

Falcao et al. [32] describes a polyphasic outbreak event in a nursery in Brazil due to multiple *Pseudomonas aeruginosa* strains. Overall, 27 children were infected or colonised by at least three different strains. One of those outbreak strains was found in an oxygen bubbler used for premature infants as well as in a rectal swab of a nurse. Screening of hands of staff was not reported. The authors assume that this nurse was the primary source of the spread of that strain via subsequent contamination of the medical device. Three other nurses were also carriers of *Pseudomonas aeruginosa* strains, but they showed different microbiological patterns and were therefore considered unrelated to the outbreak.

Passaro et al. [41] published the last outbreak report that was included in this systematic review: Seven patients in cardiovascular surgery acquired postoperative infections with a *Serratia marcescens* strain. Once again, artificial fingernails, used by a scrub nurse, were the most probable source of the outbreak. A contaminated jar of exfoliant cream at the nurse’s home served as a constant source for re-contamination of the nails. No more infections occurred when this jar had been discarded.

Moolenaar et al. [36] describe a *Pseudomonas aeruginosa* outbreak in a NICU affecting 46 patients; 16 patients deceased. HCWs were screened as a possible source and the outbreak strain was subsequently found on the hands of 2 HCWs, one of them with long artificial nails and the other one with long natural nails. A third nurse with short natural fingernails was also identified as a carrier of *Pseudomonas aeruginosa*, but this strain was not related to the outbreak.

A total of 16 postoperative wound infections from *Pseudomonas aeruginosa* were observed in an outbreak in a thoracic surgical department as mentioned by McNeil et al. [34]. The outbreak’s source could be traced to a nurse, who suffered from severe chronic (> 2 years) onycholysis of her right thumbnail. This lesion had remained unnoticed by her colleagues because it was constantly covered with flesh-colored nail polish. There were no additional cases as soon as this nurse abstained from further surgical procedures and her subsequent return to work with the previously infected thumbnail totally removed.

Taneja et al. [20] describe an outbreak in an Indian NICU caused by an enterotoxigenic *Escherichia coli* (ETEC) affecting 16 neonates. Swabs from various environmental sites and from all components of the milk feed were cultured. Staff of the dietetics department, where the reconstituted milk was prepared were also screened through the use of stool samples and swabs from hands. Besides some kitchen equipment, the hands and the stool sample of one cook was found to be ETEC positive. The outbreak terminated after his temporary removal from work for gut decontamination and enforcement of hand hygiene as well as food hygiene measures.

Zawacki et al. [31] describe a *Pseudomonas aeruginosa* outbreak on an NICU in Massachusetts. Four of the five affected infants developed pneumonia and a secondary bloodstream infection and ultimately died. Patients as potential reservoirs were ruled out. Then the infection control team screened the hands of HCWs and found five HCWs repeatedly tested positively for *Pseudomonas aeruginosa*, but only one of them carried the outbreak strain as confirmed by genotyping by pulsed-field gel electrophoresis (PFGE). The occupational health service took additional samples from other body sites and diagnosed an intermittent otitis externa colonised by this particular *Pseudomonas aeruginosa* strain.

Gupta et al. [25] report another NICU outbreak of ESBL-producing *Klebsiella pneumoniae*. Nineteen infants were infected or colonised by the outbreak strain. The identical strain was also found on the hands of two HCWs as shown by PFGE. One of those HCW wore artificial nails; the other had simply a longer nail length. Both HCWs were found to be negative in rectal swabs and urine samples. No new cases occurred after the HCWs removed the artificial nails or shortened the natural finger nails respectively.

Boszczowski et al. [27] describe an outbreak in a Brazilian NICU. Four patients were affected by the outbreak. Of those 4 patients, 3 developed a bacteremia and 1 patient a urinary tract infection. The outbreak strain was found on the hands of one assistant nurse, who suffered from onychomycosis. No new cases occurred as soon as she was removed from patient care.

Six postoperative wound infections by *Serratia marcescens* are reported from Jepson et al. [39] most probably caused by an assistant surgeon, who was unable to remove his very tightly fitted rings from his fingers. A strain indistinguishable by PFGE from the outbreak strain was cultured from that location. No further infections were noticed as soon as this HCW was requested to abstain from further operations until his rings had been removed.

Cassettari et al. [29] report nosocomial transmissions of ESBL-producing *Klebsiella pneumoniae* in an intermediate-risk neonatal unit in Brazil. The outbreak was noticed when nine patients were affected. Twenty-seven additional patients were colonised thereafter despite implementation and rigorous enforcement of numerous infection control measures. Finally, a nurse was identified who had harboured the outbreak strain (identical pulsed-field gel electrophoresis (PFGE) pattern) in onychomycotic lesions on her fingers. The outbreak terminated when she was removed from duty for topical and systemic antimicrobial therapy. Further investigation for potential intestinal carriage was considered unnecessary.

## Discussion

Newborn care in general and NICUs in particular were the main medical departments affected by outbreaks of GNB in this review. There are several other reports that add to this observation. For example, Haller et al. described a sustained outbreak of ESBL-producing *Klebsiella pneumoniae* in Bremen (Germany) in a NICU affecting 37 patients, of which 10 developed a bloodstream infection (7 fatal cases). This outbreak was detected in 2011 but retrospective analysis of charts revealed cases already back in 2009. The authors conclude that the strain must have been endemic since 2008 and that person-to-person transmission was the most likely route of transmission. Screening of 328 members of staff was performed as part of the outbreak investigation by perianal swabs. As a result, there were 293 HCW that were screened at least three times. However, the outbreak causing strain could not be detected in any of those samples; the actual source of the outbreak remained unclear [42, 45]. Of relevance, there were additional outbreaks by GNBs in German NICUs in 2010 until 2012. This accumulation of similar events finally caught the attention of the national media and public awareness which resulted in extensive screening of personnel [46].

However, screening of staff and pointing the finger on positive members may cause severe problems. Finding an outbreak pathogen on a HCW can easily lead to the feeling of guilt and stigmatisation among staff. The subsequent leave of absence, especially when unpaid, together with the fear of compensation might pose a financial challenge to the affected HCWs. These concerns make HCW screening a problematic topic and therefore a well-balanced decision between advantages and possible disadvantages should be made [1]. A more transparent discussion about all pro’s and con’s of such measures might be the best way to deal with this matter, especially if mainstream pressure from public media arises.

Decker et al. [47], conducted a case-control observational study including 400 HCW. They tested their rectal colonisation with multi-drug resistant gram-negative bacteria and vancomycin-resistant enterococci (VRE) through self-collected perirectal swabs and a questionnaire. They found that in the HCW group 4% (15/379) and in the control group 3.2% (12/376) of participants were colonised with multi-drug resistant gram-negative bacteria. This is in accordance with our data, which shows that staff is rarely the source of an outbreak with gram-negative bacteria.

However, instead of rectal screening much more attention should be paid to any unusual findings on hands of HCWs in outbreak situations. In 7 out of the 10 outbreaks in which a HCW was proven to be the source of an outbreak, the source mostly consisted of non-physiological conditions of their hands (e.g., use of artificial nails, rings or onychomycotic lesions). A careful glance of the hands of HCWs should therefore be considered before starting any extensive screening of otherwise healthy personnel on other body sites [48, 49]. Any rings should be removed before working at the patient’s site and any guidelines, in which “It is recommended that […] wedding rings may continue to be worn by ‘scrub’ and ‘non-scrub’ staff […].” should be revised accordingly [50].

The main limitation of our study is its retrospective approach. We can only rely on published outbreaks that were available by the search strategy applied as described. So there might be other relevant articles on this topic, which were missed. In addition, we lack a quality control scale and, thus, did not systematically evaluate the quality of the primary studies included in this review. Furthermore, the overall quality of outbreak descriptions still needs improvement as they often lack important information about the event [51, 52]. Thus, we herewith would like to encourage future authors and publishers to better adhere to the Outbreak Reports and Intervention Studies Of Nosocomial infection (ORION) guidelines in upcoming manuscripts [53].

## Conclusion

The data of this review shows that HCWs are extremely rarely the primary source of nosocomial outbreaks by GNB (25/1196 = 2.1%). Moreover, even if they are, it is apparent that their hands are much more important than an intestinal colonisation. Given these rather low numbers, a rectal screening of staff without signs of infection seems pointless, especially in light of the low sensitivity, high cost and discomfort for the HCW [54]. Due to the very weak evidence for the usefulness of a rectal HCW screening in identifying the source of an outbreak with GNB and the difficulties in interpreting screening results appropriately, we do not recommend HCW screening during outbreaks caused by GNB. Only in case of clear indications, e.g. GI tract infection or a strong correlation between a HCW and affected patients, should HCW screening be an option. Of course, adherence to proper hand hygiene is very much encouraged.

## Additional file

Additional file 1 Detailed table on extracted data. (XLSX 14 kb)

## Abbreviations

ESBL: extended spectrum beta-lactamase
ETEC: enterotoxigenic *Escherichia coli*
GNB: Gram negative bacteria
HCW: Healthcare worker
MRSA: Methicillin-resistant *Staphylococcus aureus*
NICU: Neonatal intensive care unit
PFGE: Pulsed-field gel electrophoresis
